# Multiple Mononeuropathy following Crotalid Envenomation: A case report

**DOI:** 10.1590/0037-8682-0273-2024

**Published:** 2024-10-28

**Authors:** Antonio Edvan Camelo-Filho, Pedro Lucas Grangeiro de Sá Barreto Lima, Francisco Luciano Honório Barreto Cavalcante, Pedro Vitor Ferreira Rodrigues, Oliver Reiks Miyajima, Pedro Braga-Neto, Paulo Ribeiro Nóbrega

**Affiliations:** 1 Universidade Federal do Ceará, Hospital Universitário Walter Cantídio, Fortaleza, CE, Brasil.; 2 Universidade Estadual do Ceará, Centro de Ciências da Saúde, Fortaleza, CE, Brasil.; 3 Centro Universitário Christus, Curso de Medicina, Fortaleza, CE, Brasil.

**Keywords:** Multiple mononeuropathy, Snake envenomation, Neuropathy

## Abstract

A 34-year-old man developed severe envenomation after being bitten by a *Crotalus durissus* (rattlesnake), which was treated with anticrotalic serum. Three weeks later, the patient reported paresthesia and neuropathic pain in the left hand, which had progressed to all four limbs. Electroneuromyography revealed asymmetric axonal sensorimotor multiple mononeuropathy. The patient was treated with prednisone, and six months later there was significant improvement in sensorimotor conduction. This is the first reported case of multiple mononeuropathy associated with *C. durissus* envenomation. Post-snake envenomation peripheral neuropathy is a rare complication requiring prompt recognition and treatment to optimize nerve function and enhance patient outcomes.

## INTRODUCTION

Ophidic accidents are a common and often overlooked worldwide health issue^1^. From 2020 to 2024, there have been 261,511 reported snakebite incidents in Brazil^2^. Of these, 3,111 have been associated with the *Crotalus durissus* species^2^, also known as South American rattlesnakes^1^ ^,^ ^3^. Due to their venomous properties, crotalid bites may present as medical emergencies that must be appropriately and urgently treated with appropriate antivenom^1^ ^,^ ^3^.

Envenomation by South American *Crotalus* species rarely causes significant local symptoms and presents with minimal or no signs at the bite site^1^ ^,^ ^3^ ^,^ ^4^. However, severe clinical manifestations can occur primarily because of the neurotoxic and myotoxic properties of the venom^3^ ^,^ ^4^. This type of envenomation has a mortality rate of 0.71%^2^, with acute renal injury being a major cause of death, often precipitated by myoglobinuria^3^ ^,^ ^4^. Neurological manifestations typically result from neuromuscular junction blockade, including ptosis, ophthalmoplegia, and acute flaccid paralysis^3^ ^,^ ^4^.

Multiple mononeuropathy (MM) is characterized by asymmetric axonal sensory and motor impairments that affect at least two distinct peripheral nerves^5^. The most common mechanism involved in MM is vasculitis of the vasa nervorum, which results in nerve injury due to ischemia^6^. Patients typically experience an acute or subacute onset of multifocal sensory deficits, muscle weakness, and pain^5^ ^,^ ^6^. MM is commonly linked to conditions, such as vasculitis, infections, or paraneoplastic syndromes^5^ ^,^ ^6^. 

Here, we report the first documented case of MM associated with *C. durissus* envenomation. After a literature review of the Cochrane Library, LILACS, SciELO, MEDLINE, PubMed, and PMC (PubMed Central) databases, no prior cases documenting this association were identified.

## CASE REPORT

A 34-year-old male was bitten on the left hand by a *C. durissus* and rapidly developed moderate pain and mild edema. In a few hours the patient progressed to a confusional state with lethargy, nausea, blurred vision, and dark urine. He was admitted to a reference center, and laboratory tests revealed elevated liver enzymes and elevated creatinine with myoglobinuria. The patient was clinically classified as severely envenomed^4^, based on the blurred vision and dark urine. Following national guidelines^4^, he was treated with anticrotalic serum produced in horses (polyclonal antibodies) at a total dose of 300 mg (recommended dosage of 20 vials^4^). The patient had a hypersensitivity reaction to the serum, manifesting as periorbital and tongue edema. He received intravenous antihistamines and corticosteroids to treat the hypersensitivity reaction with aggressive volume administration due to the risk of acute kidney injury. No neurotoxic manifestations were observed during this period, and the patient was discharged after stabilization of liver and renal functions after seven days of hospitalization with no residual symptoms. 

However, after three weeks, he presented with paresthesia and “needle-like” pain in the left hand and arm, which later progressed to the right hand. One week later, the symptoms progressed to the legs, which the patient described as a sensation of fatigue. On physical examination, the patient had an asymmetric pattern of multiple compromised nerves in a non-length-dependent pattern. This was characterized by bilateral hand weakness, graded at 4/5 on the Medical Research Council scale, affecting the ulnar and median nerve territories, with radial nerve preservation. There was no weakness in the lower limbs. Sensory examination revealed painful hypoesthesia in the hands and legs with an asymmetric pattern that was most pronounced in the bilateral ulnar nerve territories. 

Electroneuromyography (ENMG) was performed (Table 1) and revealed an axonal, subacute, sensorimotor MM in all four limbs, with ongoing signs of denervation consisting of fibrillations and positive sharp waves in the right *abductor pollicis brevis* muscle (innervated by the median nerve) and the left *first dorsal interosseous* muscle (innervated by the ulnar nerve). 

**TABLE 1:** First nerve conduction study.

Motor nerve	Segment	Latency (ms)	Amplitude (mV)	NCV (m/s)	Sensory nerve	Segment	Amplitude (µV)	NCV (m/s)
**Median**	TL	4.0/3.3	**5.7**/6.5		**Median**	D2	**6.3/3.5**	53.1/50.1
(right/left)	W-E		**4.9**/6.1	50.1/50.9	(right/left)			

**Ulnar**	TL	2.4/2.4	13.9/**6.4**		**Ulnar**	D5	**NP/NP**	-
(right/left)	W-BE		13.3/**5.0**	**46.7/48.9**	(right/left)			
	BE-AE		11.8/**5.0**	**46.5/46.2**				

**Radial**	TL	2.6/2.5	13.8/12.5		**Sup.**	D1	**6.8/3.7**	55.9/58.8
(right/left)	BE-BSG		12.8/11.3	55.1/56.7	**Radial**			
					(right/left)			

**Tibial**	TL	3.8/4.1	22.2/20.1		**Sural**	Leg	**5.9**/6.2	43.8/46.5
(right/left)	K-A		15.7/10.9	48.1/42.5	(right/left)			
	F-Wave	55.3/53.4						

					**Sup.**	Ankle	**3.9**/6.2	48.8/54.5
**Fibular**	TL	4.1/3.5	7.6/7.9	43.8/42.1	**Fibular**			
(right/left)	K-A		6.7/7.2		(right/left)			

**NCV:** Nerve conduction velocity, **TL:** terminal latency, **W:** wrist, **E:** elbow, **BE:** below elbow, **AE:** above elbow, **BSG:** Below spiral groove; **K:** knee, **A:** ankle, **Sup:** superficial; **D2:** finger 2; **D5:** finger 5, **D1:** finger 1. **NP:** No potential evoked. Bold numbers indicate abnormalities, either in absolute values or in comparison with the contralateral side (with >50% reduction). Reference values for nerve conduction studies are: i) Motor: Median nerve: NCV ≥ 50 m/s, TL ≤ 4.2 ms, Amplitude ≥ 6 mV; Ulnar nerve: NCV ≥ 50 m/s, TL ≤ 3.5 ms, Amplitude ≥ 6 mV; Radial nerve: NCV ≥ 50 m/s, TL ≤ 3.5 ms, Amplitude ≥ 5 mV; Fibular nerve: NCV ≥ 42 m/s, TL ≤ 5.5 ms, Amplitude ≥ 2 mV; Tibial nerve: NCV ≥ 42 m/s, TL ≤ 5.5 ms, Amplitude ≥ 6 mV. ii) Sensitive: Median nerve (D2): NCV ≥ 50 m/s, Amplitude ≥ 20 µV; Ulnar nerve (D5): NCV ≥ 50 m/s, Amplitude ≥ 20 µV; Superficial radial nerve (D1): NCV ≥ 50 m/s, Amplitude ≥ 15 µV; Superficial fibular nerve: NCV ≥ 42 m/s, Amplitude ≥ 5 µV; Sural nerve: NCV ≥ 42 m/s, Amplitude ≥ 6 µV. Tibial F-Wave ≤56 ms.

Extensive investigations of the potential causes of the MM were conducted. Antinuclear antibody, rheumatoid factor, anti-Ro, anti-La, C3, C4, anti-neutrophil cytoplasmic antibodies, hepatitis B surface antigen, hepatitis C virus, and human immunodeficiency virus serologies; serum protein electrophoresis; and cryoglobulins, vitamin B12, thyroid stimulating hormone, and anti-dsDNA levels were all unremarkable. The patient was treated with prednisone (1 mg/kg) and pregabalin for six months. 

The sensory issues resolved and muscle strength improved. A subsequent ENMG was performed, revealing improvements in motor and sensory amplitudes, with no signs of denervation. (Table 2).

**TABLE 2:** Second nerve conduction study.

Motor nerve	Segment	Latency (ms)	Amplitude (mV)	NCV (m/s)	Sensory nerve	Segment	Amplitude (µV)	NCV (m/s)
**Median**	TL	3.9/3.5	10.5/8.7		**Median**	D2	**7.3/4.1**	60.1/53.1
(right/left)	W-E		9.3/8.6	54.3/55.6	(right/left)			

**Ulnar**	TL	2.3/2.4	13,1/7.9		**Ulnar**	D5	**10.4/5.6**	52.5/51.7
(right/left)	W-BE		13.0/7.5	54.4/55.6	(right/left)			
	BE-AE		11.9/7.4	56.1/53.2				

**Radial**	TL	2.5/2.7	10.8/12.3		**Sup.**	D1	**5.8/3.9**	55.1/50.9
(right/left)	BE-BSG		10.5/11.6	57.1/54.7	**Radial**			
					(right/left)			

**Tibial**	TL	4.2/4.0	25.5/22.4		**Sural**	Leg	**2.3**/7.0	46.5/49.5
(right/left)	K-A		19.1/13.9	49.1/44.8	(right/left)			
	F-Wave	51.3/52.1						
								
					**Sup.**	Ankle	5.6/6.3	51.7/53.5
**Fibular**	TL		6.9/9.6	46.6/47.1	**Fibular**			
(right/left)	K-A	4.1/4.3	6.7/9.0		(right/left)			

**NCV:** Nerve conduction velocity, **TL:** terminal latency, **W:** wrist, **E:** elbow, **BE:** below elbow, **AE:** above elbow, **BSG:** Below spiral groove; **K:** knee, **A:** ankle, **Sup:** superficial; **D2:** finger 2; **D5:** finger 5, **D1:** finger 1. **NP:** No potential evoked. Bold numbers indicate abnormalities, either in absolute values or in comparison with the contralateral side (with >50% reduction). Refer to Table 1 for reference values.

## DISCUSSION

Complications from snake envenomation may arise immediately because of the venom itself or may occur later because of antivenom therapy^4^ ^,^ ^7^. Immediate complications manifest within a few hours of the bite and are attributable to the direct toxic properties of the venom. *C. durissus* venom primarily exerts myotoxic and neurotoxic effects^4^ ^,^ ^8^. Myotoxic syndromes result from hydrolases that disrupt cell membranes and degrade skeletal muscle, leading to increased permeability of the vascular endothelium^4^. This results in local symptoms, such as swelling, edema, and rhabdomyolysis. Neurotoxic effects arise from polypeptides that compete with acetylcholine for binding at presynaptic neuromuscular junctions, leading to neuromuscular paralysis characterized by symptoms, such as ptosis, ophthalmoplegia, and respiratory insufficiency^4^ ^,^ ^8^. Rarely, this venom may demonstrate procoagulant effects, as hemorrhagic manifestations are generally mild and of little intensity in cases caused by *Crotalus* in Brazil^1^ ^,^ ^4^. Delayed complications may include infections at the bite site, compartment syndromes, chronic local scars, and crotalid antivenom hypersensitivity reactions^7^ ^,^ ^8^. 

Peripheral neuropathy is an atypical feature of snake envenomation^9^. While the most potent animal neurotoxins such as those found in snake venom, primarily cause paralysis by affecting the neuromuscular junction^4^ ^,^ ^8^, cases involving exclusive damage to peripheral nerves are uncommon and poorly documented^9^ ^,^ ^10^ ^,^ ^11^. Descriptions of peripheral nervous system (PNS) involvement include focal sensory neuropathies adjacent to the bite site and Guillain-Barré syndrome (GBS)^9^ ^,^ ^10^ ^,^ ^11^.

To the best of our knowledge, this is the first reported case of MM associated with crotalid snake envenomation. It differs from previous cases of GBS because of asymmetric, predominantly sensory, non-length-dependent, sensory-motor impairment in the four limbs. GBS is an acute polyradiculoneuropathy that manifests as a rapidly evolving symmetric motor weakness beginning in the lower limbs and potentially ascending to the upper limbs and face^5^ ^,^ ^10^ ^,^ ^11^. The progression is uniform and symmetrical across both sides of the body^5^. ENMG studies of GBS have revealed patterns of demyelinating or axonal polyneuropathy^5^. In contrast, MM is characterized by damage to at least two distinct peripheral nerves, leading to asymmetric clinical manifestations^5^ ^,^ ^6^. This condition presents variably with symptoms, such as pain, sensory loss, and motor weakness, which do not adhere to a clear ascending or symmetric pattern^5^ ^,^ ^6^. In our patient, Table 1 shows a decrease in the amplitude of compound motor action potentials in the right median and left ulnar nerves, along with an asymmetric and non-length-dependent compromise of sensory nerve action potential amplitudes. 

However, the mechanisms underlying nerve injury during snake envenomation are not fully understood^8^. Immune-mediated vasculitis, due to venom^4^ ^,^ ^8^ or antivenom^7^, may explain the pattern of MM observed in this patient. A molecular mimicry mechanism between venom and myelin proteins in the PNS have been proposed for GBS after snake envenomation.^9^ This hypothesis arose from a case study of a patient bitten by a *Vipera aspis* snake who subsequently developed GBS^9^. Interestingly, despite the absence of direct PNS toxicity from the venom, the serum from the patient had specific cross-reactivity (IgM GM2 gangliosides) with several glycosylated venom proteins^9^. This observation raises the intriguing possibility that molecular mimicry, wherein venom proteins resemble PNS myelin proteins, could trigger an autoimmune response leading to acute polyneuropathy or, as in our case, MM.

Another hypothesis is that a hypersensitivity reaction to anticrotalic serum may have caused the MM. The side effects of antivenom include IgE-mediated reactions, such as angioedema and urticaria, and rarely, serum sickness^4^ ^,^ ^7^ ^,^ ^8^. Serum sickness is an immune complex-mediated reaction that typically presents as fever, rash, arthralgia, and complement activation, reflecting the host immune response to an immunogenic component of a drug^8^ ^,^ ^12^. This immune response can potentially induce nerve vasculitis, leading to MM, explaining the symptoms. However, the normal complement level and lack of fever and skin changes weakened this hypothesis in the present case. 

Snake envenomation is a common health issue worldwide, particularly in low-resource settings, such as Brazil^1^ ^,^ ^2^ ^,^ ^4^. Elusive manifestations of ophidic accidents, or even unknown side effects of serum therapy, such as MM, remain a challenge for physicians, and the entire burden of neuropathies associated with snakebites remains unknown. Prompt recognition and treatment, as demonstrated in this case, with the administration of corticosteroids and pregabalin, can significantly improve nerve function. Further studies are required to better understand the relationship between these factors and improve the quality of life of snakebite victims. 

In conclusion, this report highlights a rare case of severe crotalid envenomation from a *C. durissus* bite, leading to sensorimotor MM. Treatment with anticrotalic serum and subsequent prednisone showed notable improvement in sensorimotor function six months later. Recognition and prompt treatment of post-snake envenomation neuropathies are crucial for optimizing nerve function and enhancing patient outcomes. Further studies are required to deepen the understanding and management of this uncommon yet significant complication. 
